# Grounded cognition and the representation of momentum: abstract concepts modulate mislocalization

**DOI:** 10.1007/s00426-025-02076-6

**Published:** 2025-01-22

**Authors:** Jannis Friedrich, Markus Raab, Laura Voigt

**Affiliations:** Institute of Psychology, German Sport University Cologne, Am Sportpark Müngersdorf 6, 50933 Cologne, North-Rhine Westphalia Germany

**Keywords:** Embodied cognition, Representational momentum, Biomechanical movement, Physical invariants, Invariant representations

## Abstract

Literature on grounded cognition argues that mental representations of concepts, even abstract concepts, involve modal simulations. These modalities are typically assumed to reside within the body, such as in the sensorimotor system. A recent proposal argues that physical invariants, such as momentum or gravity, can also be substrates in which concepts can be grounded, expanding the assumed limits of grounding beyond the body. We here experimentally assessed this proposal by exploiting the representational momentum effect and the abstract concept of success. If success is grounded in the physical invariant momentum, the representational momentum effect should be larger for successful targets. We tested this hypothesis across four experiments (three pre-registered). In a surprising finding, we find hints that large trial numbers may hinder being able to find a representational momentum effect, which should be further investigated in future research. Regarding the central hypothesis, although only one experiment found statistically significant support, the effect tended toward the same direction in the three others as well. In order to draw robust conclusions about the results, we performed a mini meta, which aggregates the effects and inference statistics across the *N* = 271 participants. Across the four experiments, this effect was statistically significant, suggesting evidence in favor of the central hypothesis. These results should be interpreted with caution as there was inconsistency across experiments, suggesting the magnitude of the effect is small, and when asked who they believe moved faster, participants did not reliably indicate the successful target.

## Introduction

Considerable work in cognitive science has argued that cognition originally evolved to support goal-directed action in the environment (Anderson, 2010; Glenberg, 1997), and that even higher-level cognitive abilities such as representing abstract concepts or performing arithmetic are based on these ostensibly primitive abilities (Fischer, 2024; Glenberg & Gallese, 2012: Fischer, 2024; Friedrich et al. 2025a; Glenberg & Gallese, 2012. Within this literature, the domain of *grounded cognition* argues that the mental representations of concepts, even abstract concepts like democracy or success, involve the simulation of experience-based representations (Barsalou, 2008; Johnson, 2010; Lakoff, 2012). These are often implicitly assumed to be within the body, involving the sensorimotor, interoceptive or emotion systems. Yet, a recent proposal has argued that concepts should, theoretically, also be grounded in representations of *physical invariants*, unchanging features of physical motion such as momentum, gravity, or friction (Friedrich et al., 2024). We experimentally tested this proposal by assessing whether the abstract concept success is grounded in the representation of momentum. To do this, we exploited a classic mislocalization error whose effect size corresponds to a moving target’s momentum. If a target described as successful exhibits a larger effect, despite moving at the same speed, this provides evidence in support of success being grounded in momentum.

### Grounded cognition

Grounded cognition theories argue that mental representations of concepts consist of simulations of interaction with them (Barsalou, 2020; Reilly et al., 2023). For example, the mental representation of the verb kick consists of simulating, i.e., activating the sensorimotor system’s representation of, the action itself (Hauk et al., 2004; for review of similar findings see Pulvermüller, 2005). In line with these theories, even abstract concepts like success or democracy, which are by definition intangible and impossible to interact with using the sensorimotor system, are grounded in concrete representations (Friedrich et al, 2025a; Trumpp et al., 2024). This is supported by diverse empirical work (cf. Barsalou, 2008; Borghi et al., 2017). For example, priming old age was found to make participants slower in a lexical decision task (Macrae et al., 1998) and walking slowly made participants perceive a target as older (Mussweiler, 2006)^1^. The representation of the abstract concept old age consists (in part) of a simulation in the sensorimotor system, the activation of which shapes behavior outcomes in subsequent tasks. Concepts can also be represented via metaphoric mapping, in which abstract concepts can be represented by simulating concrete representations (Johnson, 2010; Lakoff, 2012). Consider a classic experiment assessing the abstract concept suspicion. In a series of experiments, suspicion of others lowered the threshold for participants to detect fishy smells and vice versa, smelling something fishy increased the likelihood of being suspicious of others (Lee & Schwarz, 2012). The concrete representation of a fishy smell is simulated to represent the abstract concept suspicion which subsequently alters perception. These findings demonstrate that even abstract concepts are grounded, via metaphoric mapping, in concrete representations, and these mechanisms can be detected in behavioral signatures (Friedrich et al., 2025a).

These modalities with which to represent concepts, no matter if concrete or abstract, are typically assumed to be sensorimotor, including taste and smell (Speed & Majid, 2019), interoceptive (Barsalou, 1999; Pezzulo et al., 2013; Prinz, 2012), social (Borghi et al., 2019; Borghi & Cimatti, 2009; Reinboth & Farkaš, 2022), and emotional (Kousta et al., 2011; Vigliocco et al., 2014) dimensions. These are all situated within the body. This is also reflected in the studies which investigate these grounding sources; lexical decision tasks (e.g., Papagno et al., 2009; Zwaan & Yaxley, 2003), motor interference (Glenberg & Kaschak, 2002; Villani et al., 2021), or property listing tasks (Harpaintner et al., 2018; Villani et al., 2019) all view the phenomenological source of grounding within the body.

On a short note regarding terminology: The field of grounded cognition does not have a clear nomenclature, being often called embodied cognition, situated cognition, 4E cognition, or grounded cognition (Borghi et al., 2022a; Fischer & Coello, 2016; Newen et al., 2018), with varying definitions and constraints. Aligning with past work, we argue that the term grounded cognition is the most appropriate for current purposes because it emphasizes the notion of concept grounding, and does not imply that concepts can only be grounded in the body (Friedrich et al., 2024).

### Physical invariants

Representations of physical invariants, called *invariant representations*, may also constitute a source in which to ground concepts. Humans have rich representations of physical invariants, so there is little reason to exclude them a priori, and they should theoretically qualify to be a source of grounding (Friedrich et al., 2024). This theoretical argument is tested for the first time to our knowledge in this current experiment. This is a radical departure from classical views of grounded cognition, yet in line with a more general development towards an ecological approach in grounded cognition (e.g., Borghi et al., 2022b; Kelty-Stephen et al., 2022; Rączaszek-Leonardi, 2023).

In order to qualify as a potential grounding substrate, invariant representations must not be abstract representations, but rather direct, immediate and rich. Furthermore, an important distinction within physical invariants is that it is not just the direction of the movement (the *kinematic* component), but the force underlying the movement (the *kinetic* component). As grounding in a kinematic direction (e.g., up or right) is well-evidenced in grounded cognition, critical for our theoretical proposal is that kinetic force is represented, and subsequently serves as a grounding substrate (Friedrich et al., 2024). Various work supports this. Firstly, there are dedicated perceptual features which detect forces acting on the body: the utricle and saccule, part of the vestibular system (Berthoz, 1996)^2^. Empirical work supports this further. Intuitive physics and research on physical reasoning (Kubricht et al., 2017; but see Ludwin-Peery et al., 2021) and work on internal models of gravity and other invariants (Lacquaniti & Zago, 2001; McIntyre et al., 2001; Zago et al., 2008), demonstrate that physical forces are readily simulated in service of action execution. Further, empirical work on infants suggests that physical reasoning develops very early in life, if not innate (Baillargeon, 2004; Spelke & Kinzler, 2007). One important line of research has developed around *representational momentum* (cf. Hubbard, 1995a, 2005, 2014). Here, it is argued that mental representations have, often over evolutionary timescale, integrated the representation of kinetic force into the ‘mental space’^3^. This was followed by evidence for representational gravity, -friction, and -centripetal force (Hubbard, 1995b, 2020). Multiple theories draw on this ‘internalization’ of kinetic forces: Freyd’s spatiotemporal coherence (Freyd et al., 1990) and momentum metaphor (Freyd & Finke, 1984) as well as Shepard’s second-order isomorphism (Shepard, 1984), Hubbard’s (1999) environmental invariants hypothesi, or recent advances in predictive processing's mental representations (Friedrich et al., 2025b). These latter theories and the previous empirical work all agree that invariant representations are not abstract. Rather, they are represented similarly richly, immediately, and directly as colour, direction or space, all of which are widely accepted substrates in grounded cognition (see e.g., Amsel et al., 2014; Casasanto & Bottini, 2014; Landau et al., 2010). It would seem unlikely for the mind to have such immediate representations of a modality which it neglects to use.

The theoretical proposal, first formulated in Friedrich et al. (2024), builds on this, arguing that physical invariants and the attached kinetic force are not only represented, but also capable of grounding concepts, and should therefore become part of grounded cognition theorizing. Until now, most work in grounded cognition has, implicitly, assumed that it is only possible to ground concepts in body-based representations (except the social dimension proposed by Borghi et al., 2019). No classic grounded cognition theory can even account for kinetic force or grounding in invariant representations (Friedrich et al., 2024). Our proposal is therefore heavily embedded in a rich research tradition on invariant representations, and yet poses a significant challenge to widely accepted implicit assumptions about the limits of grounded cognition. Until now this proposal has been purely theoretical, and here we test the strongest version of the proposal, that abstract concepts, via metaphoric mapping, can also be grounded in physical invariants.

### Success grounded in momentum

As we are the first, to our knowledge, to test a link between abstract concepts and invariant representations we chose a concept, success, that clearly maps onto a specific physical invariant, momentum. Why should success be grounded in momentum? Firstly, following work on conceptual metaphor theory, the metaphors used to describe success are reflective of the cognitive structures underlying their representation (Gibbs, 2005; Kövecses, 2020; Lakoff, 2012). Metaphors of success include a successful person being a “pace-setter” that is “going places” or on “the road to success”, while someone not successful may be experiencing an “obstacle to success”, or they “crash”, “lag behind”, or have “setbacks” (see Łącka-Badura, 2016). This suggests that the abstract concept success relies in part on a metaphoric mapping of forward momentum. This theoretical argument is supported by empirical findings from Robinson and Fetterman (2015). Using a joystick which is pushed forward or pulled backward, participants responded to a lexical decision task which consisted of success (e.g., “achieve”) or failure (e.g., “blunder”) words. Participants tended to be quicker to push forward for success, and backward for failure (and vice versa). Similarly, with a series of experiments Markman and Guenther (2007) argued that that psychological momentum, the uplifting experience of a series of successes, and representational momentum may share a representational substrate as they exhibit similar dynamics. Participants re-deployed lay intuitions of representational momentum to represent the current success of a given team in a basketball (study 1). Further, just as representational momentum increases with the target’s mass, psychological momentum increased with importance of the game (study 2); and that just as representational momentum carries a target forward, a person was estimated as having more success on a subsequent task after demonstrating success in prior tasks, with prior success carrying the person forward through tasks (study 3; Markman & Guenther, 2007). Conceptual metaphor theory’s theoretical postulates, combined with these empirical findings, suggest that success may be grounded in momentum, and therefore should be a valid test of the proposal of grounding in invariant representations.

### The current study

The aforementioned representation of the physical invariant momentum, called representational momentum, also gives rise to the representational momentum effect (RM effect), which makes it an excellent testbed for current purposes. The RM effect involves a moving target being suddenly occluded. Participants indicating its location at the moment of occlusion typically indicate the moving target further along in the direction of motion (Freyd, 1983; Hubbard, 2005; Kerzel et al., 2001). For example, Hubbard (1995a) had participants watch a small dot moving unidirectionally across a screen, e.g., left to right, which at random times disappeared. Participants were asked to indicate the last location of the target, and participants tended to point further along the trajectory than the actual last location (i.e., further right). A robust positive displacement has been replicated in diverse settings and experimental paradigms (e.g., Freyd & Finke, 1984; Merz, 2022; Nakamoto et al., 2015; Senior et al., 2000; for review see Hubbard, 2005).

Although the extensive past research on the RM effect has uncovered many moderators, boundary conditions and caveats (Hubbard, 2017), we introduce only those relevant for our purposes. The moderators of the RM effect roughly correspond to those factors which correspond to influences on momentum in real life. Firstly, the RM effect is moderated by the speed of the target; the faster the target, the larger the representational momentum, and correspondingly the larger the measured forward displacement (Hubbard, 2005; Merz, 2022). These moderators go beyond low-level influences and include abstract characteristics. For example, an object believed to be a rocket, which tends to move fast, elicits an RM effect, while the same object believed to be a church, which tends to not move, does not (Reed & Vinson, 1996). Past experiences and knowledge shape expectations of an object’s momentum, and this reaches into perception, shaping the size of the effect. Such research has typically focused on objectively relevant influences (i.e., a rocket really does have more momentum than a church, and it is adaptive to exploit such knowledge). This can also be interpreted as evidence of kinetic force, as opposed to kinematic direction, underlying the RM effect. The current study progresses beyond this, assessing whether the RM effect can be moderated by an objectively irrelevant characteristic, such as success.

We report here four experiments in which we tested whether a successful target produces a larger RM effect than a non-successful target. Participants first read short vignettes about two people, Nicholas and Jonathan, which portrayed them as successful and non-successful respectively. This was followed by the RM effect task. Not only did we expect an RM effect generally, but also a larger RM effect for the successful target. To triangulate possible boundary conditions of the effect we also explicitly asked participants about how they perceived the targets’ speed by asking participants to indicate which of the two persons they believed ran faster, although both had the same speed. Across all four experiments we tested the same three hypotheses: Firstly, that both targets elicit an RM effect. Second, that the successful target will more often be reported as having ran faster. Third, and most centrally, the hypothesis derived from the proposal of invariant representations, that the successful target will elicit a larger RM effect.

## Methods–experiments 1 and 2

The current experiments adopted procedures originally created by Hafri et al. (2022). Ethical approval for this study was obtained from the institutional review board of the local university (reference number 541/21). We performed a computer study with a within-subjects design with a single factor with two levels (success, non-success). In this section we include protocol and data of Experiments 1 and 2. Experiment 2 is a direct replication and did not differ in any materials or procedure. The only differences were in the sample size and that Experiment 2 was https://osf.io/fmw7y. The online repository (OSF Link) also contains documentation and files for all stimuli, code, and R scripts of all experiments.

For all experiments, most participants were students at the local university, and all spoke the native language. There has been no indication of mislocalization phenomena differing across cultures, although as grounding of abstract concepts may differ across cultures (Barsalou, 2023; Kemmerer, 2022), we note potential effects of a WEIRD sample in limiting generalizability (Henrich et al., 2010). In these experiments, there was a mean gender proportion of 33% female across all experiments.

### Materials and measurement

Participants completed these experiments in a sound-proof room in the faculty laboratories at a computer using a standard mouse and keyboard. The computer screen was an LG 24GM77-AB, 1920 × 1080px, 508 × 305 mm with a refresh rate of 144 Hz. The experiment lasted on average *M*_*exp1*_ = 15.00 min (*SD* = 3.92) and *M*_*exp2*_ = 14.21 min (*SD* = 7.31).

#### Stimuli

**Fig. 1:**
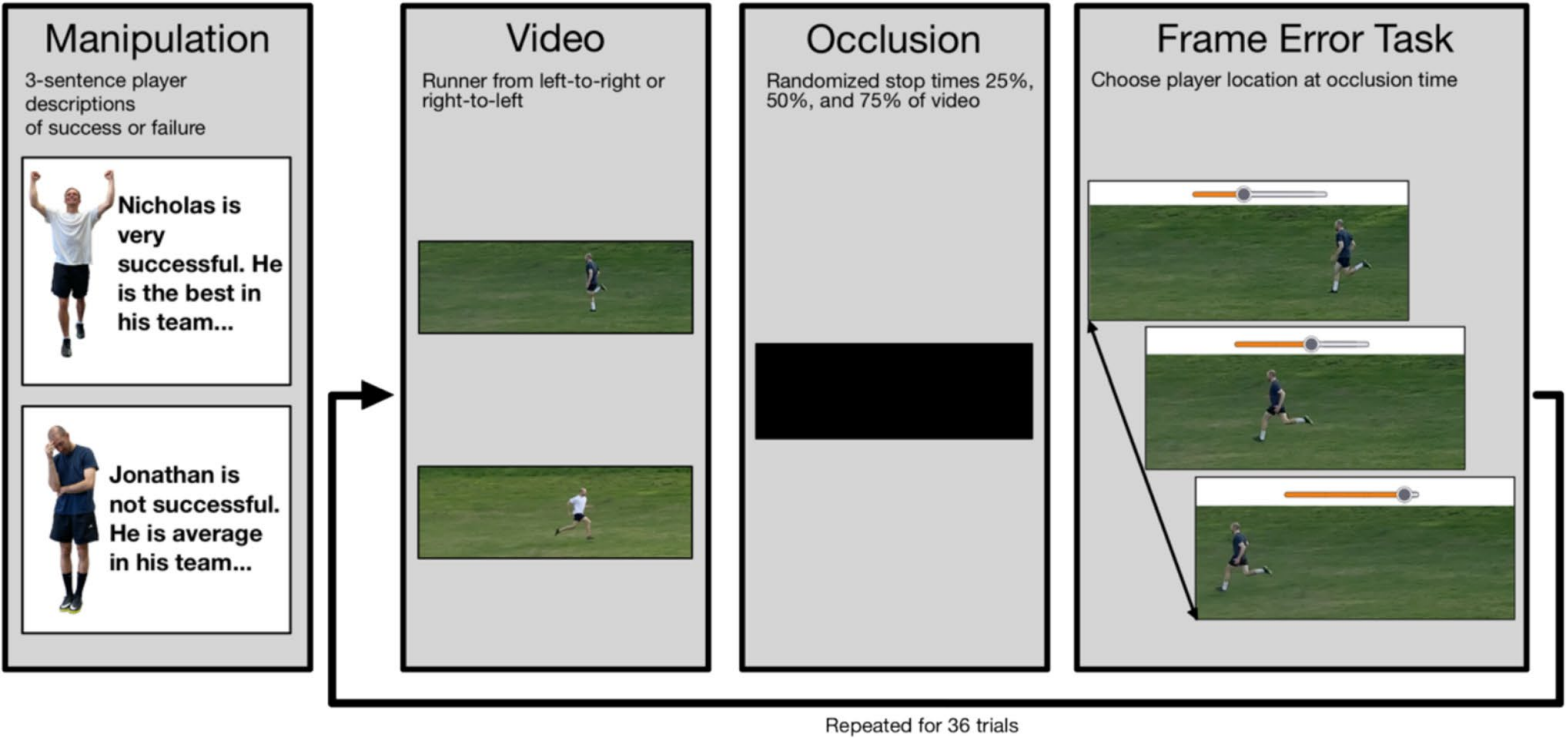
Experimental procedure of experiments 1 and 2

The experiment included twelve videos in total, each portraying one of two persons (one in black shirt, another in white, counterbalanced across participants, see Fig. 1) running horizontally across the screen. Each of these persons had three different videos running left-to-right and three running right-to-left. Including both directions ensured that there would be no confounding by motion after-effects (as in e.g., Dils & Boroditsky, 2010). Lastly, the same person was used for running in both conditions (i.e., shirt colors), portraying the different characters. This was not detected by the vast majority of participants (in debriefing only *n* = 5 participants across both experiments), and ensured to even out potential confounds in running ability or style. Background of the videos was green grass and contained no anchor points or other visual information.

All twelve videos were 3 s long, consisting of 180 frames, resulting in a frame-rate of 60fps. The length of the videos was based on the duration it took the persons to cross the screen. The frame rate provided excellent resolution. Furthermore, the target’s speed falls well within the boundaries in which the RM effect exhibits a reliable forward displacement. By having twelve different videos, we can ensure that there are unlikely to be confounds in single videos. Each video was presented randomly once at each of three pause times, resulting in 36 trials. We chose 36 trials because this would correspond to all participants seeing all combinations of video and pause time once. Videos were paused at 0.75 s (frame 45), 1.5 s (frame 90), and 2.25 s (frame 135), corresponding to 25%, 50%, and 75% of the video. These videos had been filmed from a static viewpoint, and had dimensions of 1854 × 256px. Participants sat so that their head was approximately 50 cm away from the screen. Videos were stopped with a precision of < 0.005 s variance (corresponding to less than 1 frame).

#### Manipulation of success

The manipulation consisted of portraying the two persons as either successful or unsuccessful. This was done through a vignette prior to the task, where participants read a short summary about the persons. The original German versions can be found in the online supplements to this study. We chose a moving person as the target because we felt it would be unlikely to ascribe success or non-success to an inanimate object. The summary of the successful person read:

Nicholas is often successful in life. He grew up as an outstanding student, top of his class and scoring the best in exams. Now he works at a leading company in his field and will soon get a promotion because he secured an important deal. In his free time, he seeks leisure in playing soccer and is one of the leading players in his team.

The description for the unsuccessful person read as follows:

Jonathan is not especially successful in life. He grew up as a medium student, a bit lazy in class and always scoring below average in exams. Now he works at a small company in his field and will get a demotion because he failed to secure an important deal. In his free time, he seeks leisure in playing soccer and is one of the average players in his team.

Above these descriptions were pictures of the respective persons so that participants had a clear link of the description to the color shirt which the person was wearing. Furthermore, the successful person was posed with arms up and the non-successful person in a ‘dejected’ pose, i.e., looking down with arms at the side (see Fig. 1).

#### Dependent variable

The central dependent variable was the RM effect task (based on the dependent measure from Hafri et al., 2022), shown after the video was occluded. Participants used a slider similar to those found in video players on the internet to scroll through the video frame-by-frame and select the frame which they believed was the last shown before the video was occluded. The displacement was calculated by subtracting the number of frames between the true frame (at which the video actually stopped) and the remembered frame. A larger forward displacement corresponds to a larger RM effect. Self-report of speed (for hypothesis 2) was asked directly with a binary response, “Who do you think ran faster?” with the two options “Jonathan” and “Nicholas”.

### Procedure

The experiment began with a short overview of the experiment, followed by demographic questions on age and gender. Then participants read the task instructions and did an example trial. Then the manipulation took place (see *Manipulation*, above) and finally participants performed the 36 trials in randomized order. For each trial, participants saw a photo below the video of the person who was about to run and had to press the correct name to start the video. After concluding the 36 trials, participants answered the self-report question about running speed of the two targets. Lastly, they were asked whether they used any unique strategies and what they believed was the true intention of the experiment in the debriefing.

### Data analysis

Data analysis was performed using R (RStudio Team, 2020) on R version 4.2.2 (2022-10-31). Inference criterion was an alpha level of = 0.05, Cohen’s d effect sizes, 95% effect size confidence intervals, and Bayes factors are also reported where possible. Hypothesis 1, hypothesizing a forward displacement across all trials, was tested with a one-tailed one-sample t-test on the means of participants across trials. Hypothesis 2 was tested with a one-tailed binomial test on the binary self-report item, with H_0_ = 0.5. Hypothesis 3 was tested with a one-tailed paired samples t-test on intra-condition means for each participant.

Participants were removed from analysis when they did not provide any information on gender, age, or the reported speed of the person, suggesting that they were not paying attention or not taking the experiment seriously. Following Hafri et al. (2022), we removed participants whose reported guesses on later stop times were not higher than guesses on lower times, as this would be indicative of a lack of effort. Lastly, we removed trials with extreme values, assumed at larger than *z* ≥ 3 standard deviations above or below the mean (cf. Tabachnick & Fidell, 2014). Such large deviations are unrealistic, and can therefore safely be assumed to be the result of erroneously clicking or not understanding the task.

### Transparency and openness

We adhered to the Journal Article Reporting Standards (Appelbaum et al., 2018). We reported all exclusion criteria, all manipulations, and all measures used in this study. Furthermore, as required by level 2 in the author guidelines for promoting an open research culture (Nosek et al., 2015), all materials used for this study, including the code to run the experiment, to convert data, the R Script for data preparation and analysis, all stimuli, a detailed data analysis plan and all other materials to conduct and process the study are freely available in the stable-link online repository OSF here: https://osf.io/hak4d/. The full pre-registration of Experiment 2 can be found here: https://osf.io/fmw7y. As this study was run in Javascript, HTML, and CSS, any computer with a web browser can run this study. To further ensure transparency and openness, throughout all these experiments, all deviations from pre-registration are reported (cf. Lakens, 2024; Simmons et al., 2021).

## Experiment 1

### Participants

The sample was collected using convenience sampling of *N* = 33 participants (13 female) of students at the local university. Participants were not compensated. To determine an adequate sample size was difficult as moderation effects of abstract concepts had not been done in research on mislocalization phenomena. Therefore, it was unclear whether abstract concepts have similar effect sizes on the mislocalization phenomena than physical beliefs (such as speed). Based on generally large effect sizes for moderation in other mislocalization studies (see reviews, e.g. Hubbard, 2005), we assumed a small-medium effect size (*d* = 0.45). Sample size was calculated based on this estimation using G*Power (Faul et al., 2007), yielding a desired sample size of *N* = 32, based on α = 0.05 and 80% power for a one-tailed paired sample t-test. There was *n* = 1 participant was removed based on the exclusion criteria above, for not responding to the speed question. 22 trials were removed as outliers based on the *z* ≥ 3 criterion. The final sample size used for testing was *N* = 32 (*n* = 13 female). The final sample had an average age of *M*_*age*_ = 22.56 years (*SD*_*age*_ = 2.09, min = 19, max = 28).

### Results

Regarding hypothesis 1, the overall forward displacement was *M* = 3.94 (*SD* = 2.62, 95% *CI* [3.00, 4.88]). A one-tailed one-sample t–test *t*(34) = 8.52, *p* < .001, *d* = 1.51 (95% *CI* [0.75, 2.22]), *BF*_*10*_ > 100, indicated strong evidence that there is a forward displacement. A Q-Q plot and Shapiro-Wilk normality test (*p* = .04) showed slight deviation from normality. We therefore repeated the analysis with a more robust method: A linear mixed model with forward displacement being predicted by participant-ID as a random factor included condition as a fixed factor and showed this as a significant predictor at *b* = 1.28, *se* = 0.41, *t*(1271.98) = 3.14, *p* = .002. Regarding hypothesis 2, the successful target was reported as having been faster in *n* = 20 of *N* = 32 participants (62.50%). An exact binomial test, with _0_ = 0.5 reflected this difference as not statistically significant with *p* = .11, *BF*_*10*_ = 0.93. Regarding hypothesis 3, for successful targets, the mean forward displacement was *M*_*S*_ = 4.32 (*SD* = 2.87, 95% CI [3.40, 5.47]), for non-successful targets the mean was *M*_*NS*_ = 3.45 (*SD* = 3.17, 95% CI [2.30, 4.59]), leading to an average intra-subject difference of *M*_*S−NS*_ = 0.98 (*SD* = 3.04, 95% CI [−0.11, 2.08]). A one-tailed paired-samples t-test, *t*(31) = 1.83, *p* = .038, *d* = 0.33 (95% CI [−0.16; 0.82]), *BF*_10_ = 0.83), indicated statistically significant support for hypothesis 3, that the forward displacement is larger for the successful target. Although the bayes factor suggests that this is not strong evidence, and rather evidence in favor of the null.

## Experiment 2

In line with recommendations for improving the replicability of findings and reducing the number of false-positive results (Simmons et al., 2021), we performed a pre-registered direct replication of this experiment, changing only the sample size. This was also important as the bayes factor did not provide evidence in favor of our hypothesis. There was one deviation from this pre-registration: The final sample size was *n* = 5 participants more than pre-registered. However, this does not pose an issue to its conclusions as the experiment was not at risk of being overpowered: a post-hoc sensitivity analysis based on the same values as the a priori sample size calculation (cf. below) showed that the actual achieved power was 83%.

### Participants

The sample was collected using convenience sampling of *N* = 81 participants (*n =* 32 female). Estimation of the effect size for power analyses was based on the results for hypothesis 3 in the data from Experiment 1 (*d* = 0.3). Sample size was calculated using G*Power (Faul et al., 2007), indicating a minimum sample size of *N* = 71, based on α = 0.05 and 80% power for a one-tailed paired samples t–test.

We collected *N* = 81, again accounting for dropouts, outliers, or other reasons for missing data. *n* = 5 participants were removed for not answering the self-report question about speed. 38 trials were removed for being outliers based on the *z* ≥ 3 criterion. The final sample size used for testing was *N* = 76 (*n* = 30 female; *M*_*age*_ = 29.24 years; *SD*_*age*_ = 10.93, min = 16, max = 69).

### Results

Regarding hypothesis 1, the overall forward displacement was *M* = 4.26 (*SD* = 2.81, 95% *CI* [3.61, 4.90]). A one-tailed one-sample t–test *t*(75) = 13.19, *p* < .001, *d* = 1.51 (95% *CI* [1.03; 1.98 ]), *BF*_*10*_ > 100, indicated strong evidence for hypothesis 1, that there is a forward displacement. A Q-Q plot and Shapiro-wilk test suggested no deviation from a normal distribution. Regarding hypothesis 2, the successful target was reported as having been faster in *n* = 42 of *N* = 76 participants (55.26%). An exact binomial test, with _0_ = 0.5 reflected this difference as not statistically significant with *p* = .21, *BF*_*10*_ = 0.4. Regarding hypothesis 3, for successful targets, the mean forward displacement was *M*_*S*_ = 4.31 (*SD* = 3.27, 95% *CI* [3.56, 5.06]), for non-successful targets the mean was *M*_*NS*_ = 4.20 (*SD* = 2.72, 95% *CI* [3.58, 4. 82]), leading to an average intra-subject difference of *M*_*S−NS*_ = 0.11 (*SD* = 2. 12, 95% *CI* [− 0.38, 0.59]). A one-tailed paired-samples t-test *t*(75) = 0.44, *p* = .33, *d* = 0.04 (95% *CI* [−0.28; 0.35]), *BF*_10_ = 0.14), indicating no support for hypothesis 3. On the contrary, the bayes factor suggests evidence in favor of the null hypothesis. A Q-Q plot and Shapiro-wilk test suggested no deviation from a normal distribution.

## Discussion–experiments 1 and 2

Both of these experiments found support for the RM effect (hypothesis 1). This is in line with the robustness and consistency of this effect in past literature (Hubbard, 2005, 2014; Merz, 2022). Yet, only in the first, not pre-registered experiment was the successful target reported as being faster (hypothesis 2) and exhibited a larger RM effect (hypothesis 3). Although the bayes factors of the respective studies do suggest moderate evidence in favor of the null. The two experiments and the two different statistical inference methods therefore provide contradictory conclusions. What may have been responsible?

Three central issues arose from these first two experiments. Firstly, multiple participants frequently guessed the aim of the study and they may have corrected their responses accordingly. This was evident in participants’ written comments and debriefing conversations with the investigators. They stated that, knowing we were trying to trick them into thinking the successful target was faster, they responded differently. Second, there was large error variance, and the response distributions have clear peaks in about 6 frame distances, which is the default ‘jump’ when moving the slider. Such a significant source of error variance originating from the response method could obfuscate a small effect. Third, participants reported that, having completed the information uptake and testing prior to the trials, they no longer thought about the identity of the targets after a few trials. The hypothesis that the success characteristic of the target modulates displacement, relies on this characteristic being salient during the task. In other words, if the identity of the target is irrelevant to the participants, it is unlikely that the concept of success is active, and further unlikely to affect the size of the RM effect. In order to resolve this and assess whether there is a true underlying effect of success on the RM effect, we performed a third experiment.

It is also likely that the experiments, and especially Experiment 1, was severely underpowered. Often, such ‘embodiment effects’ in which activation in the modalities affect other, ostensibly unrelated, abstract cognition (and vice versa; cf. Körner et al., 2016) involve much larger sample sizes. We therefore collect further data, which should allow to draw a robust conclusion.

## Experiment 3

We conceptually replicate the experiment with slight adjustments to address the aforementioned issues (see Table 1 for overview of changes across all experiments). This not only allows to incorporate changes from the prior experiments which may have hindered the effect, but also ensures that there is variability in the design. This ensures that there are no incidental third variables which may be responsible for the effect. Firstly, we changed the input method to a touchscreen. This meant that the error variance introduced by the fat-handed (cf. Eronen, 2020) slider which made jumps consisting of many frames was reduced by using the participant’s own finger indicating the location with precision to the exact pixel. This reduces noise in capturing of the participant’s belief about the location. It also has a much higher resolution. In one frame, it is possible that the head moved multiple pixels forward. Second, the descriptions and aim of the experiment were made more subtle so as not to arouse suspicion. This was because many participants correctly guess the true intention of the experiment, and this may have changed the reaction. Third, we also doubled the number of trials to arrive at a more accurate estimate of forward displacement. Fourth, we also aimed to degrade the visual information, which should increase reliance on the prior expectations, by making the videos shorter and heavily reducing the frame rate (as is frequently done in this field, e.g., Merz, 2022). Lastly, to strengthen the manipulation, some small changes were made to enforce more in-depth thinking about the targets’ identities and to make them more salient throughout the trials. These changes should lead to a more valid test of our core hypothesis that the RM effect is larger for the successful target.

### Methods

**Fig. 2:**
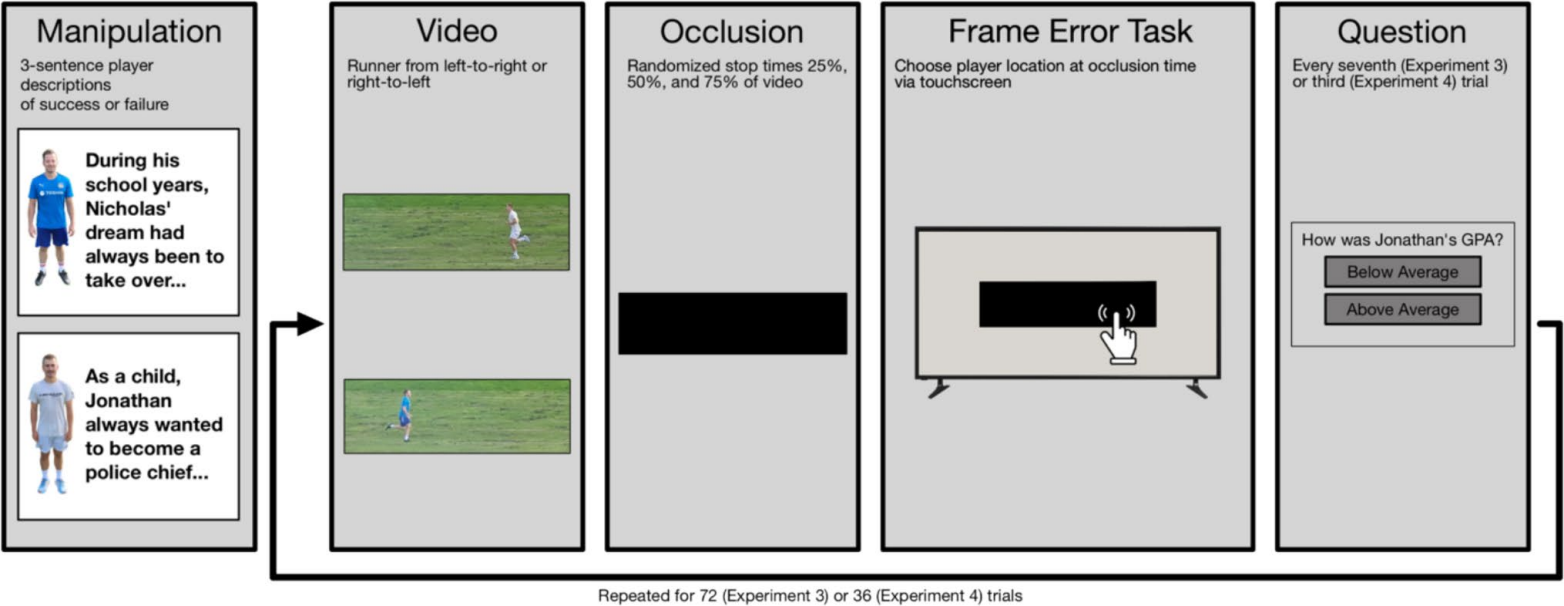
Experimental procedure of experiments 3 and 4

The third experiment retained most of the methods of Experiments 1 & 2 (see Fig. 2). Here, we only mention differences to these. This experiment was also pre-registered https://osf.io/2mbh7, and there were no deviations from the pre-registration.

### Materials and measurement

The touch screen was an iiyama PROLITE TF4338MSC-B1AG, resolution 1920 × 1080, 970 × 560 mm with a refresh rate of 60 Hz. Participants sat upright in a chair which placed their head 60 cm away from the screen. The experiment lasted on average *M*_*exp3*_ = 22.88 min (*SD* = 16.5).

#### Manipulation of success

In order to counteract participants guessing the true intention of the experiment, we had the photos of the targets not be posed and slightly changed the vignettes to be more subtle. These changes were therefore intended to prevent arousing suspicions by additionally including information that is neutral. As it could be argued that one person being described as a good soccer player evokes the impression of them being faster (by virtue of being more athletic), we have removed this aspect. This again intends to prevent an alternative third variable being an incidental cause of significant results. The vignette of the successful target now read:

During his school years, Nicholas’ dream had always been to take over his father’s company. He followed a typical school path with good to average grades. After high school, he spent half a year in Australia working as a surf instructor. After completing a degree in business administration, he familiarized himself with various aspects of his father’s company. At the age of 35, he succeeded his father as the CEO. He lives with his family, and even after 5 years, the company is doing well.

The vignette for the unsuccessful target now read:

As a child, Jonathan always wanted to become a police chief. He graduated from school with an average high school diploma and spent a year in Canada on a Work and Travel program. Afterward, he started a dual study program with the police and was later hired by them. Today, he lives alone and earns a good income in his job. However, he was perceived as too unreliable and is still far from realizing his dream of becoming a police chief.

After these descriptions, participants were asked to write one sentence which continued this description. Furthermore, participants were asked nine questions (e.g., *where did Nicholas spend his gap year?*; see online repository for all questions) about the targets interspersed throughout the RM effect task (every 7th trial). These changes should make the concept of success more salient. As participants reported no longer thinking about the targets’ attributes when performing the trials. Furthermore, the questions served as an attention check, confirming that most participants had read the descriptions carefully with an average of *M*_*correct*_ = 91.56% correct.

#### Stimuli

New videos were used which were 1 s long, consisting of 12 frames. This corresponds to a much lower fps rate of 12 (with each frame appearing for roughly 83ms). This change in frame rate and video length was done to align more with other mislocalization literature which tends to use sequences of photos to allow the participant to infer movement. We expected this degradation of visual information to lead to more reliance on internal expectations of speed and enhancing the size of the effect. Each video was presented randomly twice at each of three pause times, resulting in 72 trials. This doubling of trials was to increase power, by reducing the variability of each participant. Videos were paused at frames 5, 7, and 9, corresponding to 25%, 50%, and 75% of the video, as in Experiments 1 & 2.

#### Dependent variable

The RM effect task was now performed by participants tapping with their finger on the screen where the target’s head was at the moment the video was occluded. Participants were asked to place their hand on the arm rest as the video played, in order to prevent them moving along with the movement with their finger. The RM effect is measured as the number of pixels between the true location (i.e., where the target’s head was at occlusion time) and the indicated location in the horizontal axis. Again, a higher positive forward displacement corresponds to a larger RM effect.

### Participants

The sample was collected using convenience sampling of *N* = 80 participants (*n =* 17 female). Participants were not compensated. As pre-registered, the effect size from Experiment 2 was very small (*d* = 0.04), which would require a sample size far above our resources to detect with adequate power. Therefore, due to resource constraints (Lakens, 2022), we intended at least *N* = 65 participants to detect an effect size of approximately *d* = 0.3 as indicated in a power analysis in G*Power based on α = 0.05 and 80% power for a one-tailed paired sample t-test (Faul et al., 2007). To account for drop-outs (*n* = 5 in Experiment 2), we aimed for a sample size of *N* = 70. There was no incomplete data on the dependent variable, as no participants broke off the testing.

*N* = 9 participants were removed based on exclusion criteria, all for not writing the sentence following the vignettes. *N* = 1 participant was removed whose guessed locations on later stop times were not higher than guesses on lower times as this suggests that the participant was not trying. 45 trials were removed for being outliers based on the *z* ≥ 3 criterion. The final sample size used for testing was *N* = 70 (*n* = 17 female). The participants had an average age of *M*_*age*_ = 20.37 years (*SD*_*age*_ = 5.24, min = 15, max = 28). The final included sample size is therefore the same as pre-registered.

### Results

Regarding hypothesis 1, the overall forward displacement was *M* = 4.52 (*SD* = 24.12, 95% *CI* [− 1.23, 10.27]). A one-tailed one-sample t–test *t*(69) = 1.57, *p* = .061, *d* = 0.19 (95% *CI* [− 0.21; 0.58]), *BF*_*10*_ = 0.42, indicated no evidence for hypothesis 1. A Q-Q plot and Shapiro-wilk test suggested no deviation from a normal distribution. Regarding hypothesis 2, the successful target was reported as having been faster in *n* = 32 of *N* = 70 participants (45.71%). An exact binomial test, with _0_ = 0.5 reflected this difference as not statistically significant with *p* = .78, *BF*_*10*_ = 0.36. Regarding hypothesis 3, for successful targets, the mean forward displacement was *M*_*S*_ = 5.01 (*SD* = 24.95, *95% CI* [− 0.94, 10.96]), for non-successful targets the mean was *M*_*NS*_ = 4.03 (*SD* = 23.93, *95% CI* [− 1.68, 9.74]), leading to an average intra-subject difference of *M*_*S−NS*_ = 0.98 (*SD* = 7.97, 95% CI [− 0.92, 2.88]). A one-tailed paired-samples t–test *t*(69) = 1.03, *p* = .15, *d* = 0.04 (95% CI [− 0.29; 0.37]), *BF*_10_ = 0.22, indicated no support for hypothesis 3. A Q-Q plot and Shapiro-wilk test suggested no deviation from a normal distribution. The bayes factor of this experiment, again suggested no evidence in favor of our hypothesis 3, despite the mean difference tending to the hypothesized direction.

### Exploratory analyses

As we had doubled the number of trials to the previous experiment, and no longer found significant support for the, typically robust, RM effect, we analyzed the influence of the number of trials on response patterns. In a linear mixed model, with participant ID as a random effect, trial number significantly predicted the size of displacement such that increasing trial numbers predicted a reduction in forward displacement *β*_trial#_ = − 0.64 (*SE* = 0.08), *p *< .001. To further assess this, we repeated the hypothesis testing using only the first 36 trials. Here, statistically significant support for the RM effect (i.e., hypothesis 1) re-emerged *M* = 4.93, *t*(69) = 1.68, *p* = .04, *d* = 0.20 (95% CI [− 0.20; 0.59]), *BF*_10_ = 0.50. As well as support for hypothesis 3: The forward displacement for the successful target was *M*_*S*_ = 6.40 compared to the non-successful *M*_*NS*_ = 3.47 (difference *M*_*S−NS*_ = 2.92), *t*(69) = 1.71, *p* = .045, *d* = 0.11 (95% *CI* [− 0.22; 0.45]), *BF*_10_ = 0.52. The bayes factor in this exploratory analysis also not in favor of our hypothesis although it is more than twice as large as the one produced when taking all trials. These analyses are evidently post-hoc, were not pre-registered and should therefore be interpreted with caution.

## Discussion

This third experiment failed to provide evidence in favor of any of the three hypotheses. This is in contrast to Experiment 2, in which we failed to find evidence for hypotheses 2 and 3, but nonetheless found evidence for hypothesis 1, a forward displacement.

The mean forward displacement (hypothesis 1) was 4.52px, which corresponds to 0.22 cm, a small and non-significant displacement that is untypical in literature (Freyd, 1987; Gorman et al., 2011; Hubbard, 2005, 2017; Merz, 2022). This suggests that changes from Experiments 1 & 2 to Experiment 3 may have extinguished the RM effect generally.

Furthermore, we again found no evidence for hypothesis 3. Although once again, the effect was in the hypothesized direction, it did not reach statistical significance. This then constitutes three studies which each have mean differences in the hypothesized direction. Such a pattern is often the result of complex mechanisms underlying an effect, or very small effect sizes generally (Götz et al., 2022, 2024). Although evidence at this point is not strongly in favor of the third hypothesis, further data collection will bring the full sample size across all studies to a sample size typical for such studies, which allows us to draw robust conclusions about the phenomena.

An additional finding in exploratory analysis was that trial number was a significant predictor of the forward displacement. The forward displacement was non-significant in the full sample, but returned when analyzing only the first 36 trials, the number which the preceding Experiments 1 & 2 had used. Focus on trial number has been limited in research on the RM effect, therefore this is a novel finding (cf. general discussion). This may suggest that prior expectations only drive the forward displacement in early trials. Given this explanation, support for hypothesis 3, a difference in forward displacement between successful and non-successful targets should also only be present in these first 36 trials. Indeed, the data support this, there is a statistically significant difference in support of hypothesis 3 when taking only the first 36 trials.

## Experiment 4

In Experiment 4, we returned to having only 36 trials, in line with the theoretical background submitted by the speed prior account, and the findings of Experiments 1 & 2. We also returned to using visual stimuli similar to Experiments 1 & 2 with continuous motion. This experiment and especially its Bayes factors, suggest evidence to the contrary of the hypothesis. Although this was the third experiment in sequence in which the effect did go in the hypothesized direction. We therefore felt this warranted a follow-up study in which issues of Experiment 3 would be addressed, which would then allow to draw robust conclusions. We also identified two points that might still be hindering the effect, which were tweaked in order to more conclusively collect evidence in favor or against the effect.

First, we suspected that participants are unlikely to build a strong and lasting image of the person, when the person is switched randomly from trial to trial. This was based on participants in debriefing. We therefore blocked the trials such that in a first half participants only saw videos from one target, and in a second only the other. Second, participants in Experiment 3 still reported that the targets’ identities were no longer salient during the task. To counteract this, we made further small changes including increasing the frequency of questions from every seventh trial to every third. This has the intention of encouraging deeper processing of the characteristics and making the identity more salient.

### Methods

The fourth experiment retained the design of Experiment 3 and except for the below listed changes, all else was identical to it. This experiment was also pre-registered https://osf.io/9k4ch. There were two deviations from the pre-registration, both pertaining to the sample (cf. below). Neither reduce the validity or severity of the tests. On the contrary, they increase its severity.

### Materials and measurement

The experiment lasted on average *M*_*exp4*_ = 22.76 min (*SD* = 9.05). This is comparable to the duration of Experiment 3, in which we had 72 trials. This is due to the increase in number of questions.

#### Manipulation of success

Again, the descriptions were changed to be more in line with those of Experiments 1 & 2, emphasizing the respective success/failure (original German versions can be found in the online supplements to this study). We had attempted to make the descriptions more neutral in Experiment 3 to prevent participants from guessing the intention of the experiment. Yet it was clear that participants nonetheless guessed the intention of the experiment, while reporting that the success aspect was not very salient. The new vignette of the successful target read:

During his school years, Nicholas’ dream had always been to take over his father’s company. He followed a typical school path with very good grades. After high school, he spent half a year in Australia working as a surf instructor. After completing his degree in business administration, he familiarized himself with aspects of his father’s company, and at the age of 35 he succeeded him as CEO. He lives with his wife and two children and is very happy in his life.

This differs from the previous experiment in stating that he had very good grades and that he is very happy with his life (as opposed to the company doing well). One participant had responded previously that Nicholas was not successful in their eyes because he was not actually happy, which is why this was adjusted. The vignette for the unsuccessful target read as follows:

*“As a child*,* Jonathan always wanted to become a police chief. He graduated from school with an average high school diploma and spent a year in Canada on a Work and Travel program. Afterward*,* he started a dual study program*,* which he dropped out of*,* and later ended up in an office job. He earns enough to get by*,* but he is perceived as unreliable and is therefore far from getting a promotion. He hasn’t had much success in love and lives alone today.”*

This description differs from the previous in order to make him seem more of an unsuccessful person, by no longer even being a police officer, and his income changing from ‘good’ to ‘enough to get by’.

### Procedure

In Experiments 1–3, all videos were randomly shown in a single block. Here, they were divided into two blocks, and videos in each block only had a single person (i.e., Jonathan ran in block 1, Nicholas in block 2 or vice versa). This served to build a consistent picture of the identity across trials and make the characteristics salient. The questions which were interspersed only asked about the target running in that block. Prior to the first block and during the half time where the targets switch, participants are asked to provide two adjectives that describe the target from the upcoming block. They are also once more given the target description. This serves to encourage participants to think deeper about what the persons are like and their attributes in order to bring the concept of success into mind. The questions demonstrated, again, that participants had read the descriptions carefully, with the mean percentage per participant being *M*_*correct*_ = 97.03%.

### Participants

The sample was collected using convenience sampling of *N* = 92 participants (*n =* 25 female). As in the previous experiment, there was no limit on the smallest effect size of interest, therefore we again constrained our sample by factoring what is a feasible sample size given resources and expected effect size of previous studies (Lakens, 2022). We once again aimed to collect the same minimum sample size of *N* = 70 from Experiment 3.

A total of *n* = 3 participants were excluded. *n* = 1 participant was removed who knew the persons from the stimulus pictures personally and admitted that he could not take them seriously. Another *n* = 1 participant followed along with his hands, which was explicitly forbidden, and likely to skew the results. These were not pre-specified in the pre-registration as an exclusion criterion, but it is evident that this would reduce the validity of the test by confounding results with unrelated factors. No participants were removed based on exclusion criteria for not writing the sentence following the vignettes. *n* = 1 participant was removed whose guessed locations on later stop times were not higher than guesses on lower times as this suggests that the participant was not trying. 14 trials were removed for being outliers based on the *z* ≥ 3 criterion. The final sample size used for testing was *N* = 91 (*n =* 24 female). The participants had an average age of *M*_*age*_ = 21.24 years (*SD*_*age*_ = 5.13, min = 19, max = 33).

This sample size is *n* = 19 participants larger than that pre-registered, constituting the second deviation. This was the result of unexpected willingness on behalf of participants in convenience sampling. A sensitivity analysis revealed that this allowed to detect an effect size of *d* = 0.27 with a power of 80%, based on *p* = .05, which suggests that the study is not at risk of being overpowered. On the contrary, the increased sample size increases the severity of the test and therefore constitutes an improvement on the pre-registered methods (Lakens, 2024).

### Results

Regarding hypothesis 1, the overall forward displacement was *M* = 28.04 (*SD* = 12.65, 95% *CI* [25.40, 30.67]). A one-tailed one-sample t–test *t*(90) = 21.14, *p *< .001, *d* = 2.22 (95% *CI* [1.69; 2.72]), *BF10 *> 100, indicated strong evidence for hypothesis 1. A Q-Q plot and Shapiro-wilk test suggested no deviation from a normal distribution. Regarding hypothesis 2, the successful target was reported as having been faster in *n* = 38 of *N* = 92 participants (41.76%). An exact binomial test, with *p* = .5 reflected this difference as not statistically significant with *p* = .95, *BF10* = 0.80. Regarding hypothesis 3, for successful targets, the mean forward displacement was *M*_*S*_ = 28.80 (*SD* = 13.83, 95% *CI* [25.92, 31.68]), for non-successful targets the mean was *M*_*NS*_ = 27.27 (*SD* = 13.81, 95% *CI* [24.39, 30.15]), leading to an average intra-subject difference of *M*_*S−NS*_ = 1. 53 (*SD* = 11.14, 95% *CI* [− 0.78, 3.85]). A one-tailed paired-samples t–test *t*(90) = 1.31, *p* = .09, *d* = 0.11 (95% *CI* [− 0.18; 0.40]), *BF10* = 0.27 indicated no difference in the displacement between successful and non-successful targets. The bayes factor suggests evidence in favour of the null hypothesis, that there is no difference.

## Discussion

The findings from this experiment align with the general pattern evident in the previous three. As hypothesized, in support for hypothesis 1, the RM effect did return once the trial number was reduced to the original 36 trials. This therefore suggests an important addition to the RM effect literature. There is no support for hypothesis 2, for the third experiment in a row, which allows to draw clear conclusions.

This is not so clear for hypothesis 3, as again, the effect is in the hypothesized direction, but not quite reaching statistical significance. This pattern of repeated effects in the same direction that do not reach significance can occur when the effect under investigation is small or difficult to measure, and individual experiments therefore underpowered (Götz et al., 2024).

## General analyses

In such cases, where multiple experiments provide inconclusive results, it is recommended to perform a mini meta (Maner, 2014), a technique that, like a meta-analysis, summarizes results, but contrary to conventional meta-analyses, they are especially designed to aggregate the data in multi-experiment papers (for an introduction and primer to this technique see Goh et al., 2016). Precisely because they allow drawing for more robust conclusions than the individual significance tests, in multi-experiment papers it is recommended to “focus on meta-analytic findings rather than on individual significance tests” (Maner, 2014, p. 345).

Mini metas serve an important function for investigations of complex phenomena like cognition, which can be inconsistent (Gernigon et al., 2024; Götz et al., 2022) and therefore require larger sample sizes to reach adequate power (Cumming, 2014). Multiple experiments that are conceptually similar – and therefore measuring the same phenomenon – can together reach the necessary power to inform a conclusion about a hypothesis. This is the same principle underlying the need for adequate sample sizes, by including many participants in a single test, the test gains in validity as well as severity (Lakens, 2022, 2024). For this reason, mini metas are a critical tool for progressive and open science (Cumming, 2014; Hales et al., 2019). They strengthen transparency by encouraging transparent data reporting, reduce issues such as the file drawer problem, and improve replicability because they synthesize well-supported conclusions, and all this solely by leveraging existing data (Goh et al., 2016; Zickfeld & Schubert, 2019). To improve rigor and draw the most robust possible conclusions by aggregating the four samples into one significance test, we perform a mini meta^4^.

This mini meta provided support for hypothesis 3. P-values of the studies were combined with Stouffer’s method and report the statistical significance between all studies at *p* = .003, with a combined Cohen’s *d* = 0.27 (95% CI [0.02, 0.52]) There was no significant heterogeneity across these studies as evidenced by Cochran’s *Q* = 1.83 and *I*^*2*^ = 0.00%. To arrive at a Bayesian measure of evidence, we compare marginal likelihood ratios on this combined Cohen’s d. To do this, we specify a prior distribution with σ = 1, an uninformative prior, appropriate in our case because we have little a-priori knowledge For details of this analysis, please see the appendix. (Rouder et al., 2009). This analysis produces a bases factor in favor of our hypothesis of BF10 = 13.25. This suggests evidence in favor of the third hypothesis. We conclude that these experiments therefore provide support for hypothesis 3, that the successful target exhibits a larger displacement, despite single experiments failing to cross the conventional significance threshold of *p* = .05. It should be noted here, that the tests of a mini meta involve the standardized test statistics produced by the individual experiments, as opposed to raw dependent variables. Therefore, there are no possible issues with the change in dependent variable from Experiments 1 & 2 to 3 & 4.

The results of this mini meta may, at first, seem contrary to the results of the individual experiment. We emphasize though, that each individual experiment exhibited a trend in the expected direction (see effect sizes in Table 1). This effect did not reach significance though. The same occurs for the BFs of the individual studies which tend towards favoring the null, as with such a small effect size and few participants, there is no evidence to reject the null (i.e., in favour of it). Yet, when combining these results, the consistent, albeit small, effect can be teased out. Indeed, the effect is demonstrated quite clearly, as indicated by the substantial evidence presented by the Bayes factor. This is therefore suggestive of the presence of a small effect.

Also, it may be argued that the response on hypotheses 2 and 3 should be related: if participants perceived the target as having more momentum, they should also report them as having been faster. To assess this, we assessed whether the difference between the frame error of the successful target and of the non-successful target correlates with participants’ reporting of which target they believed ran faster. These correlations within the respective experiments are *r*_*1*_ = 0.32, *r*_*2*_ = 0.06, *r*_*3*_ = 0.07, *r*_*4*_ = − 0.41^5^.

**Table 1:** Summarizing experiments and effects

Experiment	Motion	Video Length	Trial #	Input	Blocking	Sample	Hypotheses		
							1	2	3
1	continuous	3 s	36	slider	1 block randomized	32	*p* < .001 d = 1.50	*p* = .10	*p* = .03 d = 0.33
2	continuous	3 s	36	slider	1 block randomized	76	*p* < .001 d = 1.51	*p* = .21	*p* = .33 d = 0.04
3	implied	1 s	72	touch	1 block randomized	72	*p* = .06 d = 0.19	*p* = .80	*p* = .15, d = 0.04
4	continuous	3 s	36	touch	2 blocks separate	91	*p* < .001 d = 2.21	*p* = .95	*p* = .09 d = 0.11
All (mini meta)						271	*p* < .001 d = 2.53 BF10 > 300	*p* = .089 d = 0.21 BF10 = 2.11	*p* = .002 d = 0.27 BF10 = 13.25

Colours signify significance of p-values

Green is significant at *p* = .05

## General discussion

Grounded cognition argues that the mental representations of concepts consist of simulations in modalities such as the sensorimotor system. It has recently been postulated that these modalities should be expanded to include representations of physical invariants called invariant representations (Friedrich et al., 2024). To empirically test this proposal, we assess whether abstract concepts can be grounded in invariant representations. Specifically, whether the abstract concept success is grounded in the representation of momentum, by assessing whether a target’s success modulates its perceived momentum, as measured by the size of the RM effect. We performed four experiments, of which the latter three were pre-registered.

There was clear evidence for statistically significant and large effect sizes in support of hypothesis 1, the presence of an RM effect. This aligns well with past research on mislocalizations. There was no support for hypothesis 2, that participants believed the successful target to have moved faster in any of the experiments except for the first, non-pre-registered. Lastly, hypothesis 3, larger forward displacement for the successful target, was only statistically significant in Experiment 1. Yet, across Experiments 2–4 the direction of the effect was in the hypothesized direction, approaching, but never reaching significance (*p* = .04, *p* = .33, *p* = .15, *p* = .09, for Experiments 1–4 respectively). A mini meta suggests that across experiments there is a statistically significant effect at *p* = .002, an effect size confidence interval not including zero *d* = 0.27 (95% *CI* [0.02, 0.52]), and a bayes factor of BF10 = 13.25 suggesting strong/substantial (cf. Held & Ott, 2018; Jeffreys, 1961) evidence in favor. We conclude that this constitutes evidence in support of hypothesis 3 and more generally of the proposal of grounding in invariant representations, albeit with some limitations (see below).

We replicated vast prior work on the RM effect, evidencing a forward displacement for moving targets. This effect was present and had large effect sizes as well as bayes factors that provided strong evidence in favor of its presence (BF > 300 across all participants) for Experiments 1, 2 and 4, but not in Experiment 3. We argue that the most plausible reason for the disappearance of the effect is that in Experiment 3, we doubled the number of trials compared to Experiments 1, 2, and 4, from 36 to 72. The intention was to gain a more accurate mean forward displacement for each participant, as with increased trials, random error variance should be reduced. However, this was not the case. On the contrary, a significant effect for forward displacement re-appeared when limiting data to the first 36 trials only^6^. This exemption suggests a boundary to the RM effect, which cannot be gleaned from past literature. Similar patterns of reductions in the magnitudes of effects corresponding to increasing trial numbers have recently been revealed in a variety of cognitive tasks such as lexical decision tasks (Berger et al., 2024; Miller, 2023), and our findings support a similar interpretation for the RM effect. Within mislocalization literature, this is parsimoniously explained by the *speed prior account* of the RM effect which emphasizes the role of prior expectations being integrated with current sensory experience to generate the RM effect (Merz et al., 2022, 2023). Such expectations are unlikely to be static, and it is plausible that after many trials, participants may have become accustomed to the speed, and matched their expectation to it. It would be disadvantageous for the brain to continue relying on an inaccurate internal expectation, when the repeated visual input is much more valuable. Our data therefore suggest a novel boundary condition to the RM effect, which is in line with recent findings in other domains in experimental psychology, as well as with a theoretical account of the RM effect.

We failed to find evidence throughout these experiments for hypothesis 2, that participants self-report that the successful target was faster. Even when the successful target was perceived as having more momentum as reflected in the RM effect, the target’s success status did not change how participants recall the speed of their movement. Specifically, post hoc (not pre-registered) correlational analyses between the mean difference in forward displacement between conditions and self-report of runners’ speeds are volatile across experiments (ranging from *r*_*exp4*_*=* − 0.41 to *r*_*exp1*_ = 0.32). This makes drawing a clear conclusion difficult. Nonetheless, it is possible that participants, despite perhaps perceiving the location of the target as having been further ahead, did not interpret this as the target moving faster. This, while unintuitive, is not contrary to past findings. Take for example aforementioned study by Reed and Vinson (1996) which demonstrated a larger RM effect when an object was described as a rocket compared to as a church. This study used implied motion (i.e., 4 visual cues which imply that motion is occurring), and therefore participants would not have consciously stated afterwards that either of the objects moved faster, yet nonetheless they exhibited an increased RM effect. This demonstrates that higher-level cognition may bleed into lower-level perception, but that this change may not necessarily be so strong as to travel upward again. Especially as it is not the speed which we are assessing, but rather the forward momentum. The critical component is, as mentioned the kinetic force, separate from a target’s speed. The effect size for the forward displacement is also very small, and perhaps participants were not sufficiently vigilant of their perception to notice this. Especially paired with participants’ reactance. Across experiments, *n* = 9 participants guessed the true intention of the experiment (and multiple more in informal conversations following). Multiple reported that it was obvious that the question aimed to elicit the answer that the successful target is faster and that they changed their answer accordingly. Nonetheless, this finding underlines the need for further research to disentangle these effects and generate clarity.

Hypothesis 3, that the RM effect is larger for successful than non-successful targets was only statistically significant in Experiment 1, but a mini meta aggregating all participants found statistically significant support. This support of hypothesis 3 incurs important theoretical significance for grounded cognition approaches. These typically assume that concepts gain meaning by being grounded in concrete *body*-based representations (e.g., sensorimotor, interoception), and none of the classic grounded cognition theories can account for invariant representations (Friedrich et al., 2025b). The current study, by providing first support for grounding in invariant representations therefore constitutes a heavy challenge to the established limits of grounding (Friedrich et al., 2024). Furthermore, representational momentum is best characterized as proportional to an object’s *kinetic* force, beyond just the *kinematic* direction. To our knowledge only a single unpublished experiment with mixed results (Madden & Pecher, 2007) ever investigated kinetic force. This is critical point distinguishing the invariant representations from other grounding substrates (Friedrich et al., 2024) is therefore unique support for our theoretical proposal. Lastly, we have not just found evidence supporting the notion that concepts can be grounded in invariant representations, but rather the stronger hypothesis that *abstract* concepts can be grounded in invariant representations.

An alternative explanation of our findings would argue for a body-based account. It may argue that representing success involves a metaphoric mapping not of momentum, but rather of forward movement within one’s own body, and when then viewing another person moving, this generates a larger RM effect via motor simulation. This is possible, but the effect size was largest in Experiment 1, in which participants would have constantly ‘switched’ between successful and non-successful representations (no condition showed the video for longer than 2.25 s, and participants typically responded within a second). This is more plausible under a physical invariant-, than a body-based account because perceptual changes in the body should take longer than those outside, where sudden changes are typical. Furthermore, if invariant representations are represented as the literature summarized earlier suggests, our line of argumentation is more parsimonious. A body-based account requires activation of abstract concepts to first lead to a sensorimotor activation, and in turn for this sensorimotor activation to have significant effects on the motor simulation of a target that one sees moving for such a short time. The invariant representations account requires just that the momentum which is represented to be larger because success is represented in part by representational momentum eliciting a larger RM effect. Nonetheless, it is evidently difficult to draw conclusions about this without strong inference testing, and presents a critical point of contention for future studies.

It should be addressed that the mini meta, synthesizing data from participants across all experiments, produced a small effect size (*d* = 0.27). As pre-registered, and described above, the smallest effect size of interest for us would be exceedingly small. This is because these experiments are a proof of concept: If there is any effect at all, it means that the representation of success is constituted in some part of momentum. The generated, small effect size is therefore more than satisfactory for our purposes^7^. Nonetheless one should be careful not to overstate the practical influence, as such an effect size is not likely to have significant influence for conscious perception. Nonetheless, it should also not to be undeservingly dismissed. Small effect sizes can have large implications at scale (Götz et al., 2022). For example, the RM effect is often identified in offside decisions, where it causes players to be wrongly judged as more offside (Gilis et al., 2008). At the World Cups 2002 and 2006 there were, respectively, 222 and 240 close offside decisions (Catteeuw et al., 2010). At this scale, even a small systematic influence of e.g., 5% constitutes a change of 10–12 decisions, and with a single goal often sufficing to win a game, even small systematic influences can drastically change outcomes. The same goes for the aviation (Blättler et al., 2011) or driving (Blättler et al., 2010) contexts, where even tiny differences can mean life or death.

Throughout these experiments, a significant limitation is the sample size. The repeated inability to reach statistical significance in a single experiment, yet in total finding strong evidence in favor, suggests individual experiments were underpowered. The final effect size produced by the mini meta underlines this. Although sample sizes in literature on mislocalization rarely go beyond *N* = 40 participants (e.g., Merz et al., 2023) and can be as low as *N* = 6 (e.g., Müsseler et al., 2002), research of the type we looked for here (“embodiment effects”, cf. Körner et al., 2016), frequently requires over *N* = 1000 to reach sufficient power in testing such small effect sizes (e.g., Ingendahl et al., 2021; Topolinski et al., 2022). Although in our study individual studies were underpowered (all *N *< 100), their collection into a mini meta enables a single analysis with a sample size of *N* = 271, which is still below many typical ‘embodiment effects’ studies, but enough to produce a bayes factor of BF10 = 13.25, indicating strong evidence. Therefore, the non-significance of individual experiments in this study is likely due to the individual experiments being underpowered, because the effect is small.

Lastly, the methodological variability across experiments presents a limitation. While this exploratory (cf. Sakaluk, 2016) set of studies does provide first support for a significant departure from classical grounded cognition on multiple fronts, we cannot discriminate effects within the experiments, and therefore fail to draw strong conclusions about the finer details regarding what aspects of the respective experiments enhanced or disturbed the effect. Such a discovery-oriented research approach – as we have taken – must be followed, in future studies, by a theory-testing approach (Oberauer & Lewandowsky, 2019), and therefore a vital challenge for future research is to systematically manipulate experimental factors in these experiments with large sample sizes. This will also allow to address another issue which may arise from these mixed methodologies, namely that they perhaps draw on separate cognitive processes. On the other hand, it can also be argued that the variety in experimental designs demonstrates that the effect is unlikely to be caused by undetectable third variable confounds. We used two different versions of stimuli material, two different methods of input, different descriptions, different blocking of trials and so on, and nonetheless found an effect; suggesting that the only unchanging feature (the success non-success manipulation) is likely a causal factor.

In conclusion, this study tested whether a forward mislocalization, indicative of a moving target’s perceived momentum, can be modulated by abstract concepts in four experiments. Although there is slightly mixed support, our holistic assessment of the data suggests that, indeed, success may be grounded in the representation of momentum, casting doubt onto the limits of grounded cognition, assumed to lie in the body.

## Data Availability

All raw data, source code, and analyses are available in the online repository OSF:osf.io/hak4d/ .

## Appendix

### Calculation of mini meta and bayes factor transformation

To combine the results of the individual experiments, we performed a mini meta according to Goh et al. (2016) and consequently applied a Bayes factor transformation. An R script including these calculations is available online (https://osf.io/hak4d/).

These analyses were based on the t-values (t = [1.99, 0.44, 1.03, 1.31]), corresponding degrees of freedom (df = [31, 75, 69, 90]), and p-values (p = [0.03, 0.33, 0.15, 0.09]) of the respective experiments.

From this, correlation coefficients (Pearson’s r) were calculated using the formula:

$${\text{r }} = {\text{ }}\surd \left( {{\text{t}}{\text{ }}/{\text{ }}\left( {{\text{t}}{\text{ }} + {\text{ df}}} \right)} \right){\text{ }} = {\text{ }}\left[ {0.{\text{34}},{\text{ }}0.0{\text{5}},{\text{ }}0.{\text{12}},{\text{ }}0.{\text{14}}} \right]$$

The resulting correlation coefficients were then transformed using Fisher’s z transformation:

$${\text{z }} = {\text{ }}\left( {{\text{1 }}/{\text{ 2}}} \right){\text{ }} \times {\text{ ln}}\left( {\left( {{\text{1 }} + {\text{ r}}} \right){\text{ }}/{\text{ }}\left( {{\text{1 }} - {\text{ r}}} \right)} \right){\text{ }} = {\text{ }}\left[ {0.{\text{35}},{\text{ }}0.0{\text{5}},{\text{ }}0.{\text{12}},{\text{ }}0.{\text{14}}} \right]$$

The weighted Fisher’s z mean was calculated with:

$$\overline{{{\text{z }}}} = \sum \left( {\left( {{\text{n}}_{{\text{i}}} - {\text{3}}} \right) \times {\text{ z}}_{{\text{i}}} } \right)/\sum \left( {{\text{n}}_{{\text{i}}} - {\text{3}}} \right){\text{ }} = {\text{ }}0.{\text{13}}$$

where n_i_ represents the sample sizes of the respective studies. The weighted Fisher’s z mean was then converted to a mean Pearson’s r using the inverse Fisher transformation:

$$r = (e\widehat{{{\phantom{i}}}}2\overline{z} - 1)/(e\widehat{{{\phantom{i}}}}2\overline{z} ) + 1) = 0.13$$

Which in turn produces a mean Cohen’s d across all studies of.

$${\text{d}} = \left( {{\text{2r}}} \right)/\surd \left( {{\text{1 }} - {\text{ r}}} \right) = 0.{\text{27}}$$

with a 95% confidence interval (CI) of.

$${\text{CI}} = \left[ {{\text{d }} \pm {\text{ 1}}.{\text{96 }} \times {\text{ SE}}\_{\text{d}}} \right] = \left[ {0.0{\text{2}},{\text{ }}0.{\text{52}}} \right]$$

where the standard error for Cohen’s d was derived from the Fisher’s z standard error. The p-values from the individual studies were combined using Stouffer’s method.

$${\text{Z}}\_{\text{combined}} = \sum {\text{Z}}_{{\text{i}}} /\surd {\text{k}} = {\text{3}}.{\text{14}}$$

where Z_i_ are the individual study Z-scores, and k is the number of studies. This in turn produces a combined p-value.

$${\text{p}}\_{\text{combined}} = {\text{2}} \times \left( {{\text{1 }} - {\text{ }}\Phi \left( {|{\text{Z}}\_{\text{combined}}|} \right)} \right) = .00{\text{2}}$$

where Φ represents the cumulative distribution function of the standard normal distribution. Furthermore, heterogeneity across studies was assessed using the Q and I² statistics:

$${\text{Q}} = \sum {\left( {\left( {{\text{z}}_{{\text{i}}} {\text{ }} - {\text{ }}\overline{{\text{z}}} } \right)/{\text{SE}}\_{\text{z}}^{{\text{2}}} } \right)} = {\text{1}}.{\text{87}}$$

$${\text{I}} = \left( {\left( {{\text{Q}} - \left( {{\text{k }} - {\text{ 1}}} \right)} \right)/{\text{Q}}} \right) \times {\text{1}}00\% = 0.00\%$$

### Bayesian analysis

In order to arrive at a BF10 for the results of the mini meta, we used the combined Cohen’s D.

For the prior distribution we chose σ = 1 as is standard for being very uninformative, because there is no prior information on this effect (Rouder et al., 2009). Because it is argued that this distribution favors larger effects, which would increase the value of the BF10. This prior distribution can therefore be characterized as mildly conservative for our assessment of the very small ‘embodied effects’. Therefore, following Rouder et al. (2009) and Ly et al. (2016), we used a prior centered at 0 with a scale parameter *r* = 1 / √2. This prior reflects a modest expectation of effect size variability.

The likelihoods under the respective hypotheses (H₀ and H₁) were modeled as.

$${\text{L}}\left( {{\text{H}}_{{\text{0}}} } \right) = {\text{Normal}}\left( {{\text{d}},{\text{ SE}}\_{\text{d}}} \right) = {\text{Normal}}\left( {0.{\text{28}},{\text{ }}0.0{\text{9}}} \right)$$

$${\text{L}}({\text{H}}_{{\text{1}}} ) = {\text{Normal}}({\text{d}},{\text{ }}\surd ({\text{SE}}\_{\text{d}}^{{\text{2}}} + \sigma ^{{\text{2}}} )){\text{ }} = {\text{Normal}}(0.{\text{28}},{\text{ 1}}.00)$$

Using these formulas and the observed Cohen’s d = 0.276, the Bayes factor was computed as the ratio of the marginal likelihoods:

$${\text{BF}}_{{{\text{10}}}} = {\text{L}}\left( {{\text{H}}_{{\text{1}}} } \right)/{\text{L}}\left( {{\text{H}}_{{\text{0}}} } \right) = {\text{13}}.{\text{25}}$$

## Footnotes

1 These and many similar findings are often brought into association with the replication crisis, with some critics arguing that the findings were spurious, lacked statistical rigor, or worse, the product of questionable research practices. This radical criticism has been weakened and a series of successful replications suggest that this harsh criticism may have been premature, as increasing evidence suggesting these effects are not spurious, although perhaps small (Götz et al., 2024). Especially the complexity of the mechanisms underlying cognition (cf. Gernigon et al., 2024; Sanbonmatsu & Johnston, 2019) make them fragile and unstable. Researchers may therefore struggle to uncover them, despite their existence (Baumeister, 2016). For further discussion and review of the mentioned studies and their successful replications, see Stroebe and Strack (2014).

2 Some authors have argued that representations of physical invariants originate from interaction with objects (e.g., Vicovaro, 2023). While this is an exciting debate, the theoretical proposal is agnostic towards the origin of the representation (for further disucssion see Friedrich et al., 2024), as long as the thing being represented is nonetheless a physical invariant. We will avoid a deeper discussion of this in the current paper.

3 Since the first papers on this effect, multiple different mechanisms in different frameworks and across different explanatory levels have been put forth (for an overview see Hubbard, 2010). It is also evident that there is not one single mechanism which produces forward displacements but different, interacting, components (De Sá Teixeira et al., 2019; Kerzel, 2005, 2006). Some of these argue that the forward displacement is more mechanical, such as bodily mechanisms like oculomotor overshoot (Kerzel, 2000) or the result of neuronal activation (Jancke & Erlhagen, 2010). Nonetheless, there is sufficient evidence, such as the influence of explicit abstract knowledge (Reed & Vinson, 1996) to conclude that, at least part, of the RM effect is the product of the representation of the physical invariant momentum (Hubbard, 2017).

4 The mini meta was not pre-registered.

5 this analysis was not pre-registered.

6 A statistically significant difference in support of hypothesis 3 also re-appears when limiting data to the first 36 trials.

7 We look for signatures of abstract concepts in perceptual processes. Perception is the product not of a single mechanism but many interacting components (Jolly & Chang, 2019). On the way from the higher-level cognition to perception, there are many noise factors which obfuscate this signal.
